# Typhlitis

**Published:** 1867

**Authors:** A. T. Hudson

**Affiliations:** Lyons, Iowa


*Typhlitis.* By A. T. Hudson, M.D., Lyons, Iowa.

With the hope of contributing an item that may serve to elucidate the pathology and general history of this somewhat rare disease, I am induced to report the following case:—

In consultation with a neighboring physician, I was called, Nov. 30th, to see Wm. B., an engineer, aged 37. About two months prior, he was attacked with severe pain in the right iliac region. Thinking it was probably colic or dysentery, he applied hot fomentations and took some anodyne remedies, which, in time, mitigated the severity of the distress, but they never gave complete relief. Deep-seated, throbbing pain continued, followed by swelling and tenderness, which gradually extended down the thigh, along the sciatic nerve, beyond the seat of the inflammation, and bearing some resemblance to sciatic rheumatism. Yet, from the variation and obscurity of some of the symptoms, and a brief examination by his physician, no positive diagnosis was arrived at. The difficulty of diagnosis at an early or progressive stage of the disease, is mentioned by eminent observers as very puzzling, rendering it liable to be confounded with caries of the spine, psoas abscess, or pelvic cellulitis, until recovery or a *post mortem* reveals the truth.

During the progress of the inflammation, he had become somewhat emaciated, but not prostrated; he could walk, with some difficulty; his body considerably pronated; his tongue was rather dry and brown; and his pulse varying from 110 to 120. There was much swelling above and below Poupart's ligament, which extended around, below, and behind the crest of the ilium. Here, pus had deeply burrowed under the gluteal muscles making a circumscribed tumor, with distinct fluctuation.

The discovery of pus, so remote from the seat of the pain and inflammation, caused some hesitation in pronouncing upon the diagnosis as typhlitis, yet the absence of the main features of an abscess in this part led us to conclude that it was pus from a cæcal abscess, which had traveled this way for egress—which proved correct. An incision, an inch in depth, was made before pus was reached, when a large quantity, having a strong fæcal smell, was discharged. Particles of indigestible substances were looked for, as fruit seeds, concretions, etc., but nothing found that might serve, in any way, as an exciting cause. The next day, about a pint of matter was pressed out, having the same characteristic smell and appearance. By the fourth day after opening, the discharge of pus had ceased, and there was no more escape of fæcal gas, which was noticed at previous dressings. From this time, the discharge changed to a clear, amber-colored serum, quite mucilaginous in appearance and destitute of smell, and in amount, from one to two ounces per day. On the application of heat to a portion of this fluid, it became perfectly coagulated and white, like so much pure albumen.

Being unable to find any mention of this peculiar excretion in the report of such cases as have come under my notice, I am led to believe that it is a rare occurrence. That there should be a small proportion of albumen as a component part of serum, is to be expected; but in this case, the whole of the specimen tested became cooked, leaving no appreciable part of water or other fluid behind. The sero-albuminous discharge gradually lessened, the tent removed at the end of five weeks, and the wound allowed to close, and, apparently, with a good recovery.

In about two weeks, however, after eating a hearty meal, it swelled up again, the wound reöpened, and nearly as much pus escaped as at first, and of a similar character. The amber-colored fluid succeeded the pus on the third day, and ceased in about three weeks. But little constitutional disturbance occurred from this relapse. He is now restored to health, sufficiently to attend to business.

Whether this was an abscess of the vermiform appendix, or the cæcum, or of both, is a question which cannot be clearly determined. Most authors agree that inflammation and ulceration of the vermiform appendix is always more grave and fatal than abscess of the cæcum. Dr. Bartholow, of Cincinnati, who has written an excellent review of this subject, does not give any diagnostic symptom, distinguishing one affection from the other. Probably, in every severe case, both are involved.

